# Bioactivity and Chemical Profiling of the Sea-Ice Microalga *Microglena antarctica* (Chlorophyceae)

**DOI:** 10.3390/biom15121658

**Published:** 2025-11-27

**Authors:** Riccardo Trentin, Emanuela Moschin, Luísa Custódio, Isabella Moro

**Affiliations:** 1 Department of Biology, University of Padova, Via U. Bassi 58/B, 35131 Padova, Italy; riccardo.trentin@unipd.it (R.T.); emanuela.moschin@unipd.it (E.M.); 2 Centre of Marine Sciences, Faculty of Sciences and Technology, University of Algarve, Campus of Gambelas, Ed. 7, 8005-139 Faro, Portugal; lcustodio@ualg.pt; 3 Department of Integrative Marine Ecology, Stazione Zoologica Anton Dohrn, Villa Comunale, 80121 Napoli, Italy

**Keywords:** microalgae, Antarctica, lipids, PUFA, carotenoids, antioxidants, α-amylase, tyrosinase

## Abstract

Antarctic algae have evolved in extreme environmental conditions, developing unique metabolic adaptations with significant biotechnological potential. In this study, we explored the bioactivity of the sea-ice microalga *Microglena antarctica* by preparing acetone and methanol extracts from biomass cultivated at 4, 8, and 16 °C. These extracts were screened for their in vitro antioxidant properties and inhibitory activities on enzymes related to Alzheimer’s disease (acetylcholinesterase: AChE, butyrylcholinesterase: BChE), type 2 diabetes mellitus (T2DM, α-glucosidase, α-amylase), obesity (lipase), and hyperpigmentation (tyrosinase). Our screening revealed a high capacity of acetone extracts to scavenge the ABTS•^+^ radical (EC_50_ ranging from 3.57 to 4.18 mg mL^−1^), along with strong copper chelating activity in both acetone and methanol extracts (EC_50_ values of 6.31 and 6.41 mg mL^−1^). Relevant inhibition towards α-amylase (IC_50_ values of 3.34 and 4.53 mg mL^−1^) and tyrosinase (with IC_50_ ranging from 3.82 to 5.47 mg mL^−1^) was reported for acetone and methanol extracts, respectively. UHPLC-HRMS-based profiling revealed the presence of lipidic molecules, such as glycolipids, phospholipids, and betaine lipids with polyunsaturated carbon chains, together with carotenoids, including canthaxanthin and adonixanthin, which are likely responsible for the observed bioactivities.

## 1. Introduction

Sea ice represents an important ecological niche for Antarctic microalgae, and, for its ephemeral nature, it is considered one of the most dynamic of the extreme environments on Earth [1]. This niche hosts very diverse ice-associated (sympagic) communities of organisms continually acclimating to fluctuating conditions of temperature, salinity, and light radiation and evolving through horizontal exchange and recombination of genetic material [1]. For these reasons, sea ice is considered a unique environment whose study could lead to the identification of new species, genes, and metabolites [1]. However, global warming and other environmental changes linked to human activities are seriously changing the sea-ice extent, thickness, and duration in Antarctica [2,3]. This could imply the loss of genetic and chemical diversity yet to be uncovered [1].

The study of the photosynthetic diversity in sea-ice and other Antarctic marine ecosystems is one of the goals of the Italian Antarctic Expeditions, which, through extensive sampling, have contributed to the description of several new algal species in recent years [4,5,6]. An example is *Microglena antarctica* Trentin, Negrisolo, Moschin, Veronese, Cecchetto & I. Moro, a recently described green alga with a wide distribution in Antarctica [4]. *M. antarctica* strain IMA076A was isolated from the sea ice at Inexpressible Island (Terra Nova Bay, Ross Sea), while strains ICE-L and ICE-W were isolated from the Zhongshan Research Station (Larsemann Hills, East Antarctica) [4,7]. Despite being geographically distant, these strains belong to the same species and formed a separate clade, the ‘Polar’ subclade, with the genus *Microglena* Ehrenberg emend. Demchenko, Mikhailyuk & Pröschold [4,8].

*M. antarctica* is considered the main contributor to the primary productivity in Antarctic sea-ice ecosystems and shows tolerance to extreme low temperatures, high salinity, and long-term darkness [9]. For its ecological importance and its unique adaptations to extreme environments, this species is considered an emerging model for the study of algal adaptations (morphological, physiological, biochemical, and genetic) to polar life [10,11]. The chloroplast genome of *M. antarctica* was sequenced in 2018 [9], followed by the sequencing of its complete nuclear genome in 2020 [12]. These studies offered valuable insights into the genetic adaptations of *M. antarctica* to extreme environments, showing expanded gene families involved in several processes (e.g., unsaturated fatty acid biosynthesis, DNA repair, photoprotection, ionic/osmotic homeostasis, and reactive oxygen species detoxification) and the putative acquisition of ice-binding proteins through horizontal gene transfer [12].

Despite the availability of significant genetic data, few studies on the ecology, physiology, and metabolism of *M. antarctica* are available in the literature [11,13,14,15]. Preliminary research on the photosynthetic and metabolic responses of *M. antarctica* to variations in temperature and salinity showed changes in pigment and lipid contents and profiles [11,13,14,15]. These results provide not only the first insights into stress adaptations, but also highlight the presence of valuable metabolites produced by *M. antarctica*, such as a high polyunsaturated fatty acids (PUFA) content ranging from 60% to 75% of total fatty acids [11,13,15]. The high production of PUFA is crucial for maintaining cellular membrane fluidity at low temperatures, a common survival strategy among psychrophilic species such as *Craspedostauros ineffabilis* IMA082A and *Craspedostauros zucchellii* IMA088A, which were isolated during previous Italian Antarctic expeditions [1,5,16]. These characteristics make *M. antarctica* particularly interesting from a biotechnological perspective [1,13]. In fact, PUFA are widely recognized as important molecules for human and animal nutrition and for pharmaceutical applications [17]. Along with PUFA production, the high growth rate and biomass yield observed in *M. antarctica* cultivation make this species an ideal candidate for bioprospecting [13]. Similarly, the first studies on *M. antarctica* showed the presence of highly valuable carotenoids, such as lutein, α-carotene, β-carotene, adonixanthin, canthaxanthin, and 3′-hydroxyechinenone [11,14]. These preliminary data portray *M. antarctica* as a promising candidate for bioprospecting. To date, only a few studies have investigated the bioactivity of algae isolated from Antarctica, yet with promising results [18]. Particularly, the bioprospection of green Antarctic algae has already shown high antioxidant capacities, such as in strains of *Micractinium* sp., *Chlamydomonas* sp., and *Chlorococcum* sp. [19], and skin protection properties for *Microglena antarctica* strain ICE-L [20]. Despite the lack of a policy regime on Antarctic bioprospection, the results of these explorations must be considered global public goods, as stated by the Antarctic Treaty Systems [21,22]. Specifically, article III claims that ‘scientific observations and results from Antarctica shall be exchanged and made freely available’ [22]. Thus, bioprospecting Antarctic algae holds significant potential to advance scientific knowledge and generate societal benefits.

The present study explored, for the first time, the biotechnological potential of *M. antarctica* extracts by assessing their in vitro antioxidant activity and enzyme inhibition related to human diseases. Acetone and methanol were selected as extraction solvents due to their differing polarities, which allow for the recovery of a broader range of metabolites with varying chemical characteristics. This approach aimed at maximizing the chemical diversity of the extracts and assessing potential differences in their biological activities. The extracts were obtained from algal biomass cultivated at different temperatures and further characterized by ultra-high-performance liquid chromatography coupled with high-resolution tandem mass spectrometry (UHPLC-HRMS) to annotate potential metabolites responsible for the observed biological activities.

## 2. Materials and Methods

### 2.1. Reagents

The reagents 2,2′-azinobis (3-ethylbenzothiazoline-6-sulfonic acid) (ABTS), 1,1-diphenyl-2-picrylhydrazyl (DPPH•), AChE (EC.3.1.1.7) from electrical eel, BChE (EC 3.1.1.8) from horse serum, acetylthiocholine iodide, butyrylthiocholine iodide, lipase (EC 3.1.1.3), tyrosinase (EC 1.14.18.1), glucosidase (EC 3.2.1.20), from *Saccharomyces cerevisiae*, amylase (EC 3.2.1.1) from porcine pancreas, 5,5-dithiobis(2-nitrobenzoic acid) (DBNT) and galanthamine were purchased from Sigma (Steinheim am Albuch, Germany). Other reagents and solvents were obtained from VWR International (Leuven, Belgium).

### 2.2. Cultivation and Extract Preparation

Starter cultures of *M. antarctica* strain IMA076A were grown in 100 mL of F/2 medium [23] (salinity of 35‰) at 4, 8, and 16 °C under continuous light at 35 μmol photons m^−2^ s^−1^. When the starter inocula reached the exponential growth phase, cultures were scaled up to 1 L. Cells were harvested by centrifuging at 4000× *g* for 10 min in an ALC 4236 centrifuge (ALC, Milan, Italy) at the end of the exponential growth phase and freeze-dried. Acetone 80% (*v*/*v*) and methanol 50% (*v*/*v*) extracts were obtained by mixing 0.5 g of the freeze-dried biomass with 100 mL of each solvent, respectively. Samples were disrupted with glass beads in a MM400 mixer mill (Retsch, Haan, Germany) at 30 Hz for 5 min, then incubated overnight at 4 °C. The solvent-biomass mixtures were centrifuged at 12,000× g for 10 min at 4 °C, and the supernatants were collected. The extraction procedure was repeated until the pellet became colorless, and the remaining supernatants were aliquoted into 20 mL pre-weighted vials and dried under a N_2_ stream. Extracts were redissolved in an appropriate volume of methanol to obtain stock solutions at 50 mg mL^−1^. Working solutions were prepared at a concentration of 10 mg mL^−1^ for RSA and enzymatic assays, while a 2 mg mL^−1^ extract was prepared for the determination of their chemical profiles and filtered (0.22 μm) before injection.

### 2.3. In Vitro Assays

#### 2.3.1. RSA Towards the DPPH• Free Radical

The RSA against DPPH• radical was evaluated by the method of Brand-Williams et al. [24], with modifications to adapt it to 96-well microplates. In brief, the 22 µL of extracts were mixed with 200 µL of DPPH solution (120 µM in ethanol) and incubated for 30 min at room temperature (RT) in the darkness. The absorbance was measured at 515 nm. BHT (1 mg mL^−1^) was used as a positive control.

#### 2.3.2. RSA Towards the ABTS•^+^ Free Radical

The RSA against ABTS•^+^ radical cation was evaluated as previously described by Re et al. [25], adapted to 96-well microplates. The ABTS•^+^ radical was obtained by mixing ABTS (7.4 mM) and potassium persulfate (2.6 mM) in water and incubating for 12 h in darkness at 4 °C. A working solution was then prepared by diluting the stock solution to an absorbance of 0.7 at 734 nm. In the assay, 10 µL of the extracts were mixed with 190 µL of ABTS•^+^ working solution and incubated in darkness at RT for 6 min. The absorbance was measured at 734 nm. BHT (1 mg mL^−1^) was used as a positive control.

#### 2.3.3. Ferric Reducing Antioxidant Power (FRAP)

The reduction of Fe^3+^ to Fe^2+^ by the extracts was evaluated using the method described by Megías et al. [26] in 96-well microplates. Algal extracts (50 µL) were mixed with 50 µL of 1% potassium ferricyanide, 50 µL of distilled water, and incubated at 50 °C for 20 min. Microplates were further incubated for another 10 min at 50 °C after adding 50 µL of 10% trichloroacetic acid and 10 µL of 0.1% ferric chloride. The final absorbance was measured at 700 nm, and BHT (1 mg mL^−1^) was used as a standard.

#### 2.3.4. Iron Chelating Activity (ICA)

ICA was determined according to Megías et al. [26]. Extracts (30 μL) were mixed in 96-well microplates with 200 µL of distilled water and 30 µL of an iron (II) chloride solution (0.1 mg mL^−1^ in water) and incubated for 30 min at RT. Afterwards, 12.5 µL of ferrozine solution (40 mM in water) was added, and the absorbance was measured at 562 nm. Ethylenediamine tetraacetic acid (EDTA; 1 mg mL^−1^) was used as a positive control.

#### 2.3.5. Copper Chelating Activity (CCA)

CCA was assessed in 96-well microplates as described in Megías et al. [26]. Briefly, 30 µL of the extracts were mixed with 200 µL of 50 mM sodium acetate buffer (pH 6), 6 µL of pyrocatechol violet (PV, 4 mM in the acetate buffer), 100 µL of copper sulfate (50 μg/mL in water), and the final absorbance was measured at 632 nm. EDTA (1 mg mL^−1^) was used as a positive control.

#### 2.3.6. Inhibition of Acetylcholinesterase (AChE) and Butyrylcholinesterase (BChE)

The inhibitory capacity of *M. antarctica* extracts towards AChE and BChE was evaluated using the Ellman method [27] adapted to 96-well microplates. Briefly, 20 μL of algal extracts were mixed with 140 μL of sodium phosphate buffer (0.1 mM, pH 8.0) and 20 μL of AChE or BChE solution (0.28 U mL^−1^ in sodium phosphate buffer 0.1 mM, pH 7.0) and incubated at RT for 15 min. The reaction was started by adding 10 μL of the substrates of the enzymes (acetylthiocholine or butyrylthiocholine iodide, 4 mg mL^−1^ diluted in sodium phosphate buffer 0.1 mM, pH 8.0) and 20 μL of 5,5-dithiobis(2-nitrobenzoic acid) at a concentration of 1.2 mg mL^−1^ in ethanol. After 10 min of incubation at RT, the absorbance at 412 nm was measured, and galanthamine (1 mg mL^−1^) was used as the positive control.

#### 2.3.7. Inhibition of α-Amylase

The α-amylase inhibitory activity was determined using 96-well microplates, following Xiao et al. [28]. Extracts (40 μL) were mixed with 40 μL of 0.1% starch solution (diluted in the previous buffer and 40 μL of amylase solution (100 U mL^−1^ in 0.1 M sodium phosphate buffer, pH 7.0) and incubated for 10 min at 37 °C. After that, 20 μL of 1 M hydrochloric acid (HCl) and 100 μL of iodide solution (5 mM iodine (I2) + 5 mM potassium iodide (KI), in distilled water) were added. The absorbance was measured at 580 nm, and acarbose (10 mg mL^−1^) was used as a positive control.

#### 2.3.8. Inhibition of α-Glucosidase

The baker’s yeast α-glucosidase inhibitory activity was determined in 96-well microplates according to the method described by Kwon et al. [29]. Extracts (50 μL) were mixed with 100 μL of the enzyme solution (1.0 U mL^−1^, in 0.1 M sodium phosphate buffer, pH 7.0), and incubated for 10 min at 25 °C. Subsequently, 50 μL of 5 mM p-nitrophenyl-α-d-glucopyranoside (NGP; diluted in 0.1 M sodium phosphate buffer, pH 7.0) was added, and the absorbance was measured at 405 nm after 5 min of incubation at 25 °C. Acarbose (10 mg mL^−1^) was used as a positive control.

#### 2.3.9. Inhibition of Lipase

The inhibitory activity on porcine lipase was evaluated according to the method of McDougall et al. [30] adapted to 96-well microplates. Algal extracts (20 μL) were mixed with 200 μL of Tris-HCl buffer (100 mM, pH 8.2), 20 μL of the enzyme solution (1 mg mL^−1^ in Tris-HCl buffer), 20 μL of the substrate (4-nitrophenyl dodecanoate, 5.1 mM in ethanol), and incubated for 10 min at 37 °C. The final absorbance was read at 410 nm, and orlistat (1 mg mL^−1^) was used as the positive control.

#### 2.3.10. Inhibition of Tyrosinase

The inhibitory activity against tyrosinase was determined in 96-well microplates using the method reported by Zengin [31]. Briefly, the extracts (70 μL) were mixed with 30 mL of the enzyme (333 U mL^−1^ in phosphate buffer, pH 6.5) and incubated for 5 min at RT. Afterwards, 110 μL of the substrate (L-tyrosine, 2 mM in water) was added and incubated for 30 min at RT. The final absorbance was measured at 492 nm, and arbutin (1 mg mL^−1^) was used as the positive control.

### 2.4. Ultra Performance Liquid Chromatography-High Resolution Mass Spectrometry (UHPLC-HR-MS) Analysis

*M. antarctica* extracts were profiled with a Thermo Scientific™ UltiMate™ 3000 UHPLC coupled with an Orbitrap Elite (Thermo Fisher Scientific, Waltham, MA, USA) mass spectrometer equipped with a Heated Electro-Spray Ionization source (HESI-II; Thermo Scientific). Briefly, 5 μL of extracts were separated with a Thermo Scientific Accucore RP-18 column (2.1 × 100 mm, 2.6 μm) with 40 min runs. The binary mobile phases consisted of ultra-pure LC-MS grade water (phase A) and LC-MS grade acetonitrile (phase B), both with 0.1% formic acid. Both gradient and acquisition parameters are reported in Silva et al. [32]. UHPLC-HR-MS data were acquired with Xcalibur v4.1 Qual Browser (Thermo Scientific). MS-DIAL v5.3 was used for feature finding, alignment, and annotation [33] after checking the optimal parameters for the analyses. Lipid annotation was performed automatically using in silico LipidBlast v68 [34] spectral libraries, while carotenoids were annotated using the ESI(+)-MS/MS from standards+bio+in silico library available at https://systemsomicslab.github.io/compms/msdial/main.html#MSP (accessed on 5 November 2024). The results were manually curated with the confirmation of the characteristic product ions and neutral losses to reduce false positives.

### 2.5. Statistical Analysis

The results of both antioxidant and enzymatic assays were analyzed using one-way ANOVA, followed by Tukey’s tests for pairwise comparisons, after verifying data normality and homogeneity of variances in R version 3.5.3. When the biological activities of the extracts (at 10 mg mL^−1^) were above 50%, maximal effective concentrations (EC_50_ mg mL^−1^) and the half-maximal concentration values (IC_50_ mg mL^−1^) were calculated. Results were expressed as mean ± standard deviation (SD), and experiments were conducted using six replicates.

## 3. Results

### 3.1. Antioxidant Activity and Enzymatic Inhibition

The highest antioxidant activities in *M. antarctica* extracts were observed in the ABTS and CCA assays, while the extracts showed moderate to low RSA towards DPPH, FRAP, and ICA (Table 1). The lowest EC_50_ values for ABTS•^+^ were observed in the acetone extracts from biomass grown at 16 °C (3.57 mg mL^−1^), and 8 °C (3.89 mg mL^−1^), followed by 4 °C (4.18 mg mL^−1^) (Table 1). The highest CCA was observed in the methanol and acetone extracts with values of 6.41 and 6.31 mg mL^−1^, respectively (Table 1). No significant differences were found between the CCA of methanol and acetone extracts from biomass cultivated at 8 °C.

Acetone extract from *M. antarctica* showed strong inhibition capacity towards α-amylase, with IC_50_ values of 3.34 mg mL^−1^ and 4.53 mg mL^−1^ at 8 °C and 4 °C, respectively (Table 2). Methanol extracts from biomass cultivated at all tested temperatures showed high inhibitory activity on tyrosinase, with IC_50_ values of 3.82, 4.62 and 5.47 mg mL^−1^ at 16 °C, 8 °C and 4 °C (Table 2). The extracts showed moderate to low inhibitory activities towards AChE, BChE, α-glucosidase, and lipase.

### 3.2. Chemical Profiling

#### 3.2.1. Glycolipids

The glycolipids annotated included monogalactosyldiacylglycerol (MGDG) and digalactosyldiacylglycerol (DGDG) and were observed in negative spectra as [M+HCOO]- ions, with their characteristic ions in MS/MS (253.1 and 235.1 *m*/*z*) (Table 3) [17].

#### 3.2.2. *Phospholipids*

The phospholipids annotated in *M. antarctica* extracts in negative ion mode as [M − H]- ions included lysophosphatidylglycerol (LPG), with 153.0 and 171.0 *m*/*z* typical ion fragments, and lysophosphatidylinositol (LPI), with 241.0 and 223.0 *m*/*z* as characteristic ion fragments (Table 4) [17].

#### 3.2.3. Betaine Lipids

The betaine lipids identified in this study in positive ion mode as [M+H]+ ions were lysodiacylglyceryl-N,N,N-trimethyl-homoserines (LDGTS), showing a typical fragment ion of 144.1 *m*/*z* (Table 5) [17].

#### 3.2.4. Carotenoids

The carotenoids observed in *M. antarctica* extracts (Table 6) in positive ionization mode were canthaxanthin (565.40 *m*/*z*; 547.39, 203.14 ms^2^) and adonixanthin (583.41 *m*/*z*; 565.40 ms^2^), 3″-hydroxyechinenone (567.42 *m*/*z*; 549.40 ms^2^) and 4-ketotorulene (549.41 *m*/*z*; 531.39, 427,29 ms^2^). A feature with 699.41 *m*/*z* was annotated in silico as a carotenoid, but no spectral information on this molecule is available in the literature.

## 4. Discussion

The biological activities of methanol and acetone extracts of *M. antarctica* cultivated at three temperatures were assessed in vitro for the first time using a combination of well-established antioxidant and enzymatic assays to explore the biotechnological potential of this species. In terms of antioxidant capacity, acetone extracts from *M. antarctica* showed interesting scavenging activity on ABTS•^+^ with EC_50_ values ranging from 3.57 to 4.18 mg mL^−1^. However, these values are higher than those previously reported in the lipid-rich extracts from mesophilic green commercial microalgae, such as *Dunaliella salina* (EC_50_ of 0.82 mg mL^−1^), *Scenedesmus obliquus* (EC_50_ of 0.29 mg mL^−1^), *Tetraselmis chui* (EC_50_ of 0.41 mg mL^−1^), *Chlorella vulgaris* (EC_50_ of 0.51 mg mL^−1^), *Chlorococcum amblystomatis* (EC_50_ of 0.53 mg mL^−1^), and *Nannochloropsis oceanica* (EC_50_ of 1.02 mg mL^−1^) [35,36]. These differences might be interspecific and/or due to the different culturing conditions and extraction methods [37]. In particular, the previously mentioned microalgal extracts evaluated for RSA on ABTS•^+^ [35,36] were obtained using the Folch’s method, which is specifically designed for lipid extraction [38]. This lipid-specific approach is generally more efficient than the extraction methods used in the present study (acetone and methanol) and may result in a higher yield of bioactive lipids in the extracts, which are likely responsible for the observed RSA [17]. Since metal chelators can act as antioxidants by binding to metal ions that catalyze radical generation, the extracts were further evaluated for their capacity to chelate iron and copper ions [39]. *M. antarctica* extracts exhibited Cu^2+^ chelating activity, ranging from 33.45% to 61.59%, with the highest values observed in both acetone and methanol extracts (EC_50_ values of 6.31 and 6.41 mg mL^−1^, respectively) obtained from algae cultivated at 8 °C. Other species of green algae are known for their copper-chelating abilities, including *Desmochloris* sp. SBL3 and *Nannochloris* sp. SBL1, which demonstrated chelation efficiencies of 61% and 45%, respectively, in methanol extracts at a concentration of 10 mg mL^−1^ [40]. However, the green microalgae explored for Cu^2+^ chelating activity by Pereira et al. were mesophilic species cultivated under different conditions of light intensity, temperature, and photoperiod [40]. The observed antioxidant activities might be related to the presence of algal lipids, as reported in other green algae, such as *Chlorococcum amblystomatis*, *D. salina*, *Nannochloropsis granulate*, *Neochloris oleoabundans*, *T. chui*, and *Scenedesmus obliquus* [35,40,41,42]. Other compounds with antioxidant activities, such as carotenoids, were detected in *M. antarctica* extracts. The most abundant were canthaxanthin and adonixanthin, two poorly studied ketocarotenoids, whose presence and antioxidant functions have been reported in other species of green algae such as *Chlorella* spp., *Chlorococcum* spp., *Coelastrella* spp., *Scenedesmus obliquus,* and *Tetraedron minimum* [43].

The inhibition of enzymes involved in relevant human disorders was tested for *M. antarctica* extracts. The results indicate a significant inhibitory activity against α-amylase and tyrosinase, two enzymes of pharmaceutical, cosmetic, and nutraceutical importance. Specifically, α-amylase inhibition is widely used to manage hyperglycemia in type 2 diabetes mellitus, as this enzyme catalyzes the hydrolysis of dietary starch and sugars into absorbable glucose [44]. Similarly, tyrosinase inhibition, as this enzyme catalyzes key steps in melanin biosynthesis, plays a strategic role in the treatment of hyperpigmentation disorders and in preserving food quality [45]. The acetone extracts from the algal biomass obtained at 4 and 8 °C displayed IC_50_ values of 4.53 mg mL^−1^ and 3.34 mg mL^−1^, respectively, for α-amylase inhibition. Although promising, the results obtained from *M. antarctica* extracts showed limited inhibition of α-amylase compared to highly active extracts from brown seaweed rich in phlorotannins, which exhibited IC_50_ values of 0.045 mg mL^−1^ in *Ascophyllum nodosum* and of 0.059 mg mL^−1^ in *Fucus vesiculosus* [46]. The enzymatic inhibitory properties detected in *M. antarctica* extracts might be related to other algal compounds, such as lipids and carotenoids [47,48]. Oleic acid, palmitic acid, and linoleic acid, fatty acids found in the complex lipid chains of *M. antarctica*, have demonstrated IC_50_ values of 0.97 mg mL^−1^, 0.39 μg/mL, and 0.23 μg/mL, respectively, against α-amylase and have been suggested as potential antidiabetic [47,48]. However, the mechanism of action of these molecules needs to be elucidated [49]. The presence of these fatty acids in *M. antarctica* was confirmed by GC-MS analysis using FAME standards [11]. Methanol extracts of *M. antarctica* showed high inhibitory activity on tyrosinase, with IC_50_ values ranging from 3.82 to 5.47 mg mL^−1^. Extracts from various phylogenetic groups of algae have already demonstrated significant in vitro inhibitory activity against tyrosinase, with IC_50_ values ranging from 0.009 to 0.027 mg mL^−1^, and in vivo studies have confirmed their potential as promising candidates for industrial and biotechnological applications [50]. The main class of tyrosinase inhibitors described in algae consists of phenolic compounds, with *Ecklonia stolonifera* being the most extensively studied species for the presence of phloroglucinol and its derivatives [51]. Although no phenolic compounds have been reported in *M. antarctica* extracts, other compounds (e.g., the lipids identified in this study) might have contributed to the inhibitory activity towards tyrosinase. Recently, lipid extracts ranging from 0.100 to 0.800 mg mL^−1^, derived from the thraustochytrid *Schizochytrium limacinum* and the dinoflagellate *Crypthecodinium cohnii*, were shown to reduce tyrosinase activity in a concentration-dependent manner [52]. The author suggested that fatty acids (i.e., saturated and polyunsaturated) might be responsible for the tyrosinase inhibition [52]; however, further investigation is needed to elucidate the compounds responsible for the observed activity.

The majority of compounds annotated in *M. antarctica* extracts were lipids, primarily belonging to the classes of glycolipids (MGDG and DGDG), phospholipids (LPG, PE, LPI, and LPA), and betaine lipids (LDGTS). In particular, MGDG and DGDG are abundant constituents of the thylakoid membranes of algae and were detected in both *M. antarctica* strain IMA076A and strain ICE-L [11,14]. Nevertheless, the lipid composition of *M. antarctica*, and of Antarctic algae in general, remains largely unexplored [11,14]. Algal lipids have recently attracted significant attention due to their antimicrobial, anti-tumoral, antioxidant, and anti-inflammatory properties, as well as for the presence of polyunsaturated fatty acids (PUFAs) in their fatty acyl chains, which are essential for human and animal nutrition [17,53]. The other group of metabolites annotated in *M. antarctica* extracts was carotenoids, lipophilic pigments with well-known antioxidant, anti-inflammatory, anti-aging, and anti-tumoral properties, which can also protect against cardiovascular diseases, diabetes, cataracts, and neurodegeneration [54]. Thus, as mentioned above, these metabolites could be responsible for the biological activities measured in *M. antarctica*.

The observed chemical profiles, and consequently the biological activities of the extracts, are likely to be influenced by both the extraction solvents and the cultivation temperatures. Acetone, in particular, proved more effective than methanol for extracting certain lipid classes, such as glycolipids [55]. Regarding temperature, our results show differences in biological activity between extracts obtained from cultures grown at different temperatures, suggesting temperature-dependent shifts in metabolite production. This observation is consistent with earlier studies: for instance, *M. antarctica* ICE-L showed increased PUFA and glycolipid content under cold stress, while strain IMA076A displayed higher carotenoid content at elevated temperatures [11,14]. These patterns suggest temperature modulates specific metabolic pathways, likely as an adaptation to maintain membrane fluidity and/or enhance photoprotection [11,14]. Although our UHPLC-HR-MS analysis was qualitative, the observed differences in biological activity between extracts support the hypothesis that both solvent and temperature affect the metabolite profile. Future targeted and quantitative studies are needed to confirm these variations and identify which metabolic pathways are up- or down-regulated under different cultivation conditions. Overall, the results obtained in this study highlight the potential of *M. antarctica* as a valuable source of natural bioactive lipids and carotenoids, representing a novel species with applications in the food, feed, cosmetic, and pharmaceutical industries. Since the crude extracts obtained from *M. antarctica* contain complex mixtures of metabolites, future research should focus on fractionation and compound isolation to identify the bioactive molecules responsible for the observed biological activities.

## 5. Conclusions

*M. antarctica* is a recently described species with the potential to serve as a model organism for cold-adapted algae and as a source of valuable compounds. The present study revealed the antioxidant capacity of *M. antarctica* extracts and their inhibitory activities on both tyrosinase and α-amylase. This microalga was characterized by the presence of glycolipids, phospholipids and betaine lipids, molecules known for their wide range of biological activity. These lipidic molecules were esterified with PUFAs, a characteristic that renders *M. antarctica* suitable for the nutraceutical and feed industry. Another class of biologically active compounds reported in the *M. antarctica* extract was carotenoids, such as canthaxanthin and adonixanthin, whose biological activities require further in-depth investigation. Further analyses are therefore necessary to explore other potential properties of *M. antarctica* extracts (e.g., anti-inflammatory and anti-tumoral), to quantify their metabolites, and to assess the presence of other classes of valuable molecules (e.g., peptides, proteins, phenols, terpenes, polysaccharides).

## Figures and Tables

**Table 1:** RSA towards DPPH• and ABTS•^+^ radicals, ferric reducing antioxidant power (FRAP), and metal-chelating activities on copper (CCA) and iron (ICA) of acetone and methanol extracts from *M. antarctica* cultivated at 4, 8, and 16 °C. Results are expressed as antioxidant activity (% activity) at the concentration of 10 mg mL^−1^ and as half maximal effective concentrations (EC_50_, mg mL^−1^) for extracts displaying an activity above 50%. Values are presented as mean ± standard deviation (SD), N = 6. The letters next to the values in the table indicate significantly different groups for each column (Multiple Comparisons of Means: Tukey’s HSD, 95% family-wise confidence level).

		DPPH	ABTS	ABTS EC_50_	FRAP	CCA	CCA EC_50_	ICA
**4 °C**	**Acetone**	21.36 ± 1.31 b	75.59 ± 4.24 d	4.18 ± 0.21 c	29.86 ± 4.17 b	45.12 ± 0.72 c		26.76 ± 2.81 c
	**Methanol**	13.47 ± 1.18 a	41.82 ± 4.53 b		26.24 ± 12.04 b	44.53 ± 2.01 c		27.23 ± 2.01 c
**8 °C**	**Acetone**	32.85 ± 2.45 c	83.59 ± 3.63 e	3.89 ± 0.20 bc	21.51 ± 4.41 ab	58.10 ± 1.24 b	6.31 ± 0.62 b	25.13 ± 2.37 bc
	**Methanol**	11.32 ± 1.09 a	50.84 ± 3.55 c		19.39 ± 5.65 ab	61.59 ± 4.00 b	6.41 ± 1.82 b	26.69 ± 4.65 c
**16 °C**	**Acetone**	38.73 ± 2.64 d	90.67 ± 6.21 ef	3.57 ± 0.09 b	49.49 ± 14.51 c	40.44 ± 1.73 c		15.27 ± 1.37 a
	**Methanol**	10.83 ± 1.14 a	23.35 ± 2.80 a		10.26 ± 2.93 a	33.45 ± 1.26 a		20.29 ± 1.48 ab
**BHT**		91.16 ± 1.17 e	93.20 ± 1.38 f	0.12 ± 0.01 a				
**EDTA**						95.50 ± 0.54 d	0.18 ± 0.03 a	91.16 ± 1.17 d

**Table 2:** Enzymatic inhibitory properties of acetone and methanol extracts from *M. antarctica* cultivated at 4, 8, and 16 °C. Results are expressed as inhibitory activity (% of inhibition) at the concentration of 10 mg mL^−1^ and as half maximal inhibitory concentration (IC_50_, mg mL^−1^) for extracts displaying an activity above 50%. Values are presented as mean ± standard deviation (SD), N = 6. The letters next to the values in the table indicate significantly different groups for each column (Multiple Comparisons of Means: Tukey’s HSD, 95% family-wise confidence level).

		AChE	BChE	α-Glucosidase	α-Amylase	α-Amylase IC_50_	Tyrosinase	Tyrosinase IC_50_	Lipase
**4 °C**	**Acetone**	11.36 ± 4.88 a	35.03 ± 4.11 d	11.62 ± 2.50 a	95.73 ± 6.62 c	4.53 ± 0.29 c	48.31 ± 0.70 a		30.53 ± 1.44 b
	**Methanol**	16.16 ± 3.15 ab	16.82 ± 5.53 ab	19.89 ± 1.84 b	nd		69.57 ± 1.22 b	5.47 ± 0.58 c	26.92 ± 5.97 ab
**8 °C**	**Acetone**	14.06 ± 5.29 ab	19.52 ± 4.71 ab	36.82 ± 1.41 d	109.55 ± 1.85 c	3.34 ± 0.26 b	46.55 ± 1.014 a		30.62 ± 1.80 b
	**Methanol**	21.53 ± 3.33 b	25.11 ± 3.63 bc	33.48 ± 4.15 cd	nd		88.30 ± 1.56 d	4.62 ± 0.49 bc	13.76 ± 3.58 a
**16 °C**	**Acetone**	11.33 ± 8.21 a	13.46 ± 3.69 a	18.65 ± 1.412 b	42.76 ± 4.25 a		49.52 ± 2.73 a		40.96 ± 7.24 c
	**Methanol**	30.84 ± 3.99 c	33.35 ± 4.82 cd	29.01 ± 1.63 c	nd		76.59 ± 1.09 c	3.82 ± 0.50 b	27.40 ± 8.42 ab
**Galanthamine**		84.47 ± 0.58 d	85.67 ± 1.37 e						
**Acarbose**				83.85 ± 1.52 e	57.18 ± 8.70 b	0.42 ± 0.03 a			
**Orlistat**									70.49 ± 1.87 d
**Arbutin**							91.35 ± 1.37 d	0.26 ± 0.02 a	

**Table 3:** Annotated glycolipids in negative [M+HCOO]- ion mode. MGDG, monogalactosyldiacylglycerol; DGDG, digalactosyldiacylglycerol; (−) absent, (+) present.

Rt (min)	*m*/*z* Value	Annotated Metabolite	Adduct	Formula	16 °C Acetone	16 °C Methanol	8 °C Acetone	8 °C Methanol	4 °C Acetone	4 °C Methanol
**34.65**	741.48016	MGDG 30:3|MGDG 14:0_16:3	[M+HCOO]-	C39H68O10	+	−	+	−	+	−
**33.66**	739.46454	MGDG 30:4|MGDG 14:0_16:4	[M+HCOO]-	C39H66O10	+	−	+	−	+	−
**35.65**	769.51099	MGDG 32:3	[M+HCOO]-	C41H72O10	+	−	+	−	+	+
**34.63**	767.49585	MGDG 32:4|MGDG 16:1_16:3	[M+HCOO]-	C41H70O10	+	−	+	−	+	−
**33.56**	765.47968	MGDG 32:5|MGDG 16:2_16:3	[M+HCOO]-	C41H68O10	+	−	+	−	+	−
**32.94**	763.46484	MGDG 32:6|MGDG 16:3_16:3	[M+HCOO]-	C41H66O10	+	−	+	−	+	−
**31.96**	761.44812	MGDG 32:7|MGDG 16:3_16:4	[M+HCOO]-	C41H64O10	+	−	+	−	+	−
**30.87**	759.43414	MGDG 32:8|MGDG 16:4_16:4	[M+HCOO]-	C41H62O10	+	−	+	+	+	+
**35.12**	799.55817	MGDG 34:2|MGDG 16:0_18:2	[M+HCOO]-	C43H78O10	+	−	−	−	+	−
**37.25**	797.5423	MGDG 34:3|MGDG 18:1_16:2	[M+HCOO]-	C43H76O10	+	−	+	−	+	−
**35.50**	795.52661	MGDG 34:4|MGDG 16:2_18:2	[M+HCOO]-	C43H74O10	+	−	+	−	+	−
**34.83**	793.51154	MGDG 34:5|MGDG 18:2_16:3	[M+HCOO]-	C43H72O10	+	−	+	+	+	+
**33.87**	791.49646	MGDG 34:6|MGDG 16:3_18:3	[M+HCOO]-	C43H70O10	+	−	+	−	+	−
**32.92**	789.4787	MGDG 34:7|MGDG 18:3_16:4	[M+HCOO]-	C43H68O10	+	−	+	−	+	−
**34.37**	825.57391	MGDG 36:3|MGDG 18:1_18:2	[M+HCOO]-	C45H80O10	+	−	−	−	+	−
**35.74**	821.54205	MGDG 36:5|MGDG 18:2_18:3	[M+HCOO]-	C45H76O10	+	−	+	−	+	−
**35.02**	931.56567	DGDG 32:3	[M+HCOO]-	C47H82O15	+	−	+	−	+	−
**33.46**	929.54871	DGDG 32:4	[M+HCOO]-	C47H80O15	+	−	+	−	+	−
**31.53**	927.53149	DGDG 32:5	[M+HCOO]-	C47H78O15	+	−	+	−	+	−
**30.65**	925.52118	DGDG 32:6	[M+HCOO]-	C47H76O15	+	−	+	−	+	−
**29.51**	923.5014	DGDG 32:7|DGDG 16:3_16:4	[M+HCOO]-	C47H74O15	+	−	+	−	+	−
**28.46**	921.48505	DGDG 32:8	[M+HCOO]-	C47H72O15	+	−	+	−	+	−
**33.77**	957.58032	DGDG 34:4|DGDG 16:2_18:2	[M+HCOO]-	C49H84O15	+	−	+	−	+	−
**33.10**	955.56403	DGDG 34:5|DGDG 18:2_16:3	[M+HCOO]-	C49H82O15	+	−	+	−	+	−
**31.83**	953.54889	DGDG 34:6	[M+HCOO]-	C49H80O15	+	−	+	−	+	−

**Table 4:** Annotated phospholipid in negative [M−H]- ion mode. LPG, lysophosphatidylglycerol; PE, phosphatidylethanolamine; LPI, lysophosphatidylinositol; LPA, lysophosphatidic acid; (−) absent, (+) present.

Rt (min)	*m*/*z* Value	Annotated Metabolite	Adduct	Formula	16 °C Acetone	16 °C Methanol	8 °C Acetone	8 °C Methanol	4 °C Acetone	4 °C Methanol
**26.343**	483.2731	LPG 16:0	[M−H]-	C22H45O9P	+	+	+	+	+	+
**25.807**	481.25751	LPG 16:1	[M−H]-	C22H43O9P	+	+	+	+	+	+
**26.939**	509.28879	LPG 18:1	[M−H]-	C24H47O9P	+	+	+	+	+	+
**25.42**	507.2738	LPG 18:2	[M−H]-	C24H45O9P	+	+	+	+	+	+
**24.331**	505.25842	LPG 18:3	[M−H]-	C24H43O9P	+	+	+	+	+	−
**24.775**	571.28925	LPI 16:0	[M−H]-	C25H49O12P	+	+	+	+	+	+
**22.098**	565.24268	LPI 16:3	[M−H]-	C25H43O12P	+	+	+	+	+	+
**21.331**	563.22723	LPI 16:4	[M−H]-	C25H41O12P	+	+	+	+	+	+

**Table 5:** Annotated betaine lipid in positive [M+H]+ ion mode. LDGTS, lysodiacylglyceryl-N,N,N-trimethyl-homoserine; (−) absent, (+) present.

Rt (min)	*m*/*z* Value	Annotated Metabolite	Adduct	Formula	16 °C Acetone	16 °C Methanol	8 °C Acetone	8 °C Methanol	4 °C Acetone	4 °C Methanol
**26.924**	472.36111	LDGTS 16:1	[M+H]+	C26H49NO6	+	+	+	+	+	+
**25.227**	470.34564	LDGTS 16:2	[M+H]+	C26H47NO6	+	+	+	+	+	+
**24.409**	468.33014	LDGTS 16:3	[M+H]+	C26H45NO6	+	+	+	+	+	+
**23.317**	466.31412	LDGTS 16:4	[M+H]+	C26H43NO6	+	+	+	+	+	+
**27.628**	498.3768	LDGTS 18:2	[M+H]+	C28H51NO6	+	+	+	+	+	+
**26.126**	496.36139	LDGTS 18:3	[M+H]+	C28H49NO6	+	+	+	+	+	+
**22.143**	494.34488	LDGTS 18:4	[M+H]+	C28H47NO6	+	+	+	+	+	+

**Table 6:** Annotated carotenoids in positive [M+H]+ and [M+Na]+ ion mode; (−) absent, (+) present.

Rt(min)	*m*/*z* Value	Annotated Metabolite	Adduct	Formula	16 °C Acetone	16 °C Methanol	8 °C Acetone	8 °C Methanol	4 °C Acetone	4 °C Methanol
31.43	567.4167	3″-Hydroxyechinenone	[M+H]+	C40H54O2	+	+	+	−	+	+
31.79	549.4075	4-Ketotorulene	[M+H]+	C40H52O	+	+	+	+	+	+
31.99	583.4119	Adonixanthin	[M+H]+	C40H54O3	+	+	+	+	+	+
32.01	565.4025	Canthaxanthin	[M+H]+	C40H52O2	+	+	+	+	+	+
33.57	699.4197	Unkown catonenoid	[M+Na]+	C42H60O7	+	+	+	−	+	−

## Data Availability

Data are available from the corresponding author upon reasonable request.
